# Epidemiological profiles of SARS-CoV and SARS-Cov-2 in Singapore and its promising containment strategies

**DOI:** 10.7189/jogh.11.03027

**Published:** 2021-01-30

**Authors:** Rini Chaturvedi, Sumit Malhotra, Amit Sharma

**Affiliations:** 1 Molecular Medicine Group, International Centre for Genetic Engineering and Biotechnology, New Delhi, India; 2 Centre for Community Medicine, All India Institute of Medical Sciences, New Delhi, India

In 2002, a viral outbreak emerged from China (Guangdong province) which caused severe acute respiratory syndrome (SARS) that affected 29 countries with a total of 8422 cases and 916 deaths over the period of ten months (November 2002-August 2003) [1]. This outbreak was caused by an *Orthocoronovirinae* virus of the *Coronaviridae* family and was later termed as SARS-CoV. The disease caused by SARS-CoV was considered to be the first severe and readily transmissible outbreak in the 21st century [1]. The most SARS affected countries in 2002-2003 were China followed by Canada and Singapore. The transmission of SARS-CoV to Singapore occurred when a few tourists visited the country from Hong Kong and spread the virus [1]. In the SARS-2003 outbreak, the total number of cases reported in Singapore were 238 with the first case reported on 25 February 2003 to the last case reported on 5 May 2003 [1]. Singapore was able to contain the viral outbreak within four months of its inception.

In December 2019, Wuhan in China reported a similar *Orthocoronavirinae* outbreak due to a novel coronavirus (SARS-CoV-2) causing the disease later termed COVID-19 [2]. SARS-CoV-2 spread rapidly across the globe within two months and was declared as a pandemic in March 2020 by WHO [3]. As of 8 December 2020, 216 countries have reported a total of >65 million confirmed cases with 1.5 million deaths due to COVID-19 [3]. The SARS-CoV-2 is similar to SARS-CoV virus since both are caused by *Orthocoronavirinae* belonging to the same family – *Coronaviridae* with the same betacoronavirus genus. The primary reservoir for SARS-CoV and SARS-CoV-2 is considered to be bats (**Table 1**) [4]. The two coronaviruses share 86% identity of their whole genomes and the spike protein of both viruses target the same receptor angiotensin-converting enzyme 2 (ACE-2) for their entry into the human’s respiratory tract (**Table 1**) [5,6]. SARS-CoV and SARS-CoV-2 share similar risk factors of disease outcomes in old-aged patients, and the severity of the infection also depends on underlying conditions/co-morbid illnesses. The transmissibility pattern of COVID-19 is far more severe than SARS, as the latter was able to infect over 8000 cases and was contained over the period of ten months while COVID-19 infected over 80 000 confirmed cases in only two months [7]. The reproductive number (R0) for SARS-CoV-2 (2-2.5) is higher than that of SARS-CoV (1.7-1.9) so SARS-CoV-2 might be more capable of efficient and rapid transmission (**Table 1**) [8]. Further, COVID-19 patients are reported with mild symptoms that contribute to its transmission potential as the cases might go unnoticed. One major difference between these two viruses is that SARS-CoV-2 can also be transmitted by asymptomatic patients which was not observed in SARS-CoV infections (**Table 1**) [9]. A recent report suggests that the viral load of SARS-CoV-2 detected in asymptomatic patients was similar to that of mild-symptomatic patients while lower than the severe diseased individuals [9]. Thus, the efficiency of transmission of SARS-CoV-2 asymptomatic patients contributes much higher transmissibility rate than SARS-CoV.

**Table 1:** Similarities and differences between SARS-CoV and SARS-CoV-2

Features	SARS-CoV	SARS-CoV-2
Country and year of Origin	Guangdong, China (November 2002)	Wuhan, China (December 2019)
Primary reservoir	Bats	Bats
Intermediate reservoir	Palm Civets	Remains unclear
Spike protein target Receptor	Angiotensin-Converting enzyme-2 (ACE2)	Angiotensin-Converting enzyme-2 (ACE2)
R_0_	1.7-1.9	2-2.5
Incubation period	2-7 days	1-14 days
Period of infection with active cases	Ten months (November 2002-August 2003)	Ten months and ongoing (December 2019-to date)
Transmission	Aerosols/Droplets, contact with infectious individuals	Same
Transmission by Asymptomatic carriers	No	Yes
Prevention	Hygiene, social distancing, face masks	Same
Total no of cases (Singapore)	238 (February 2003-May 2003)	58 285 (23 January-8 December 2020)
Total no of deaths (Singapore)	33 (February 2003-May 2003)	29 (23 January-8 December 2020)
Case fatality rate (Singapore)	14%	0.05%

**Figure:**
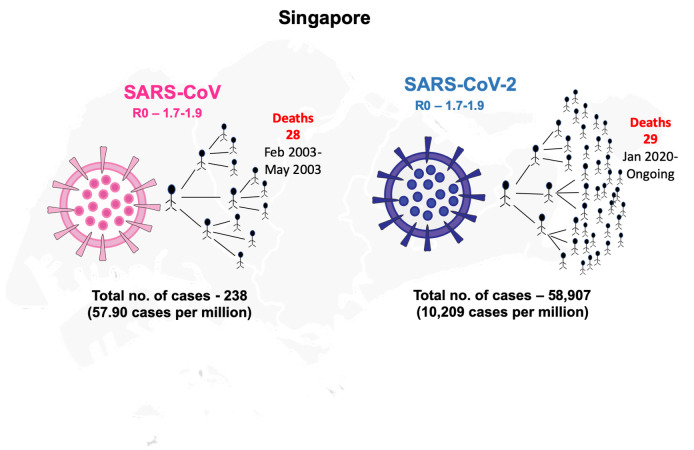
Photo: From the author’s own collection, used with permission.

## VARIATION IN TRANSMISSION RATES OF CORONAVIRUSES AND INFLUENZA VIRUS

**Figure 1:**
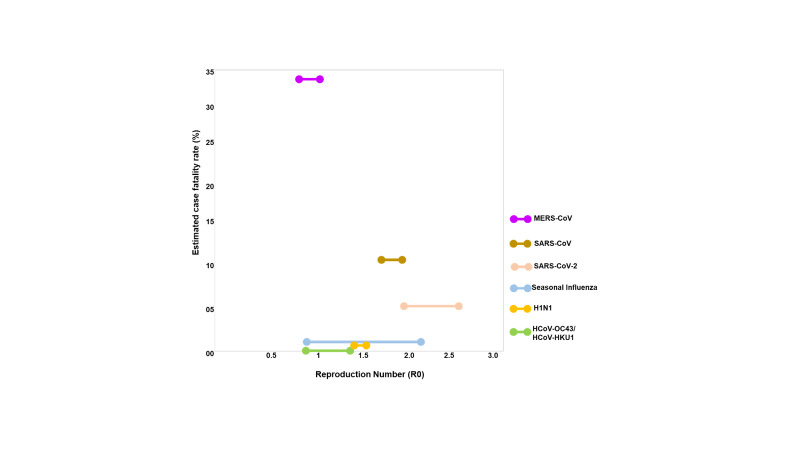
Comparison of the reproduction number (R0) and the estimated case fatality rates (%) of various human infecting coronaviruses and influenza virus. The range for reproduction number (R0) is plotted against the estimated case fatality rate (%) on the y-axis. The case fatality rate for both H1N1 and seasonal influenza is low ( ~ 0.1%-0.2%) while SARS-CoV and MERS have high fatality rates of 10% and 34% respectively. The case fatality rate for SARS-CoV-2 was calculated by using the formula (number of confirmed deaths/total number of confirmed cases) × 100.

To understand the transmissibility and the severity of SARS-COV-2, we analysed the transmission rate and case fatality rates of the human coronaviruses and influenza viruses (**Figure 1**). SARS-CoV, SARS-CoV-2, MERS (Middle-East Respiratory Syndrome) and the other two coronaviruses (HCoV-OC43 and HCoV-HKU1) belong to the same betacoronavirus genus. R0 for HCoV-OC43 and HCoV-HKU1 is ~ 1 and both are responsible for causing either asymptomatic or mild cases of respiratory tract infections (**Figure 1**) [10]. The R0 values for SARS-CoV-2 is highest among the other contagious viruses (SARS-CoV, MERS, and H1N1) that have caused several epidemics/pandemics within two decades (**Figure 1**). As of 8 December 2020, the current current estimated fatality rate globally for SARS-COV-2 is 2.3% which is still considerably lower than SARS-CoV (10%) and MERS (34%) (**Figure 1**). The high R0 value of SARS-CoV-2 is thus supportive of the fact that SARS-CoV-2 has the highest transmission rates amongst other respiratory tract targeting viruses.

## SINGAPORE’S EFFECTIVE APPROACH TO LIMIT SARS INFECTION

**Figure 2:**
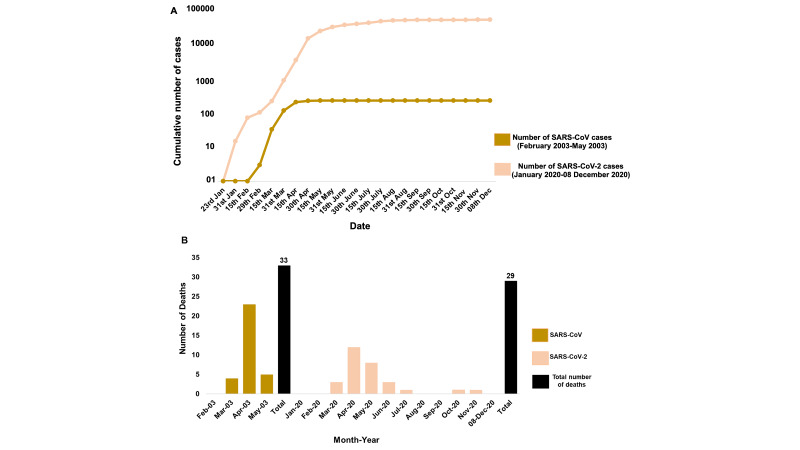
Comparison of the number of cases and deaths for SARS-CoV (2003) and SARS-CoV-2 (2020) in Singapore. **Panel A.** Cumulative number of cases reported for both SARS-CoV and SARS-CoV-2. Note that for purpose the cases for SARS 2003 outbreak are shown till August although the last case was reported for the same in May 2003). **Panel B.** The total number of deaths caused by SARS-CoV in four months in 2003 was 33, while 29 deaths have been reported due to SARS-CoV-2 from January to 8 December 2020. The last COVID-19 death in Singapore was documented on 28 November 2020 after one month from October 2020.

The highly effective strategy of Singapore in identifying the initial cases and back tracing the new cases to the initial ones is noteworthy. In SARS-2003 outbreak, Singapore divided the infection cases into four clusters and linked all probable cases to five super-spreaders and thus effectively contained the spread of the virus within four months (February-May 2003). The highest case peak for SARS-CoV was observed in the months of March and April (>100 cases in each month) which showed a steep decline in May 2003 (**Figure 2****,** Panel A) [1]. Singapore was then removed from the WHO list of areas with local transmission and was listed as SARS-free in June 2003 [1]. The highest reported individuals were from the health care segment ( ~ 41%) and family members ( ~ 24%) [11]. For COVID-19 outbreak, Singapore utilised similar techniques of linking the new probable cases to pre-symptomatic transmission [12]. The first case of SARS-CoV-2 infection was reported on 23 January 2020. Early transmission was primarily caused by imported cases and the infection rate remained low till mid-March [11]. The government of Singapore first divided the probable cases into three epidemiological clusters (a tour group from China, a company conference, and a church) to link and quarantine the contacts of initial cases till February [13]. The epidemiological clusters were later increased to seven when local transmission began from mid-March 2020 [12]. From late March and April 2020, multiple dormitories with foreign workers were included in the clusters which then contributed to a sharp rise in the number of local transmission cases in April to mid-May (**Figure 2****,** Panel A). To counteract the increasing number of cases from April, the Singapore Ministry of Health announced tighter measures of “circuit breaker period” from mid-April till early June 2020. The rigorous actions taken by the government for isolation, surveillance, and containment strategy led to a declining phase of infections in the country by mid-June. As of 08th December 2020, the cumulative number of cases from January has reached to 58 285. But interestingly out of these 58 285 total cases, 58 176 have already been recovered and only 80 cases are active at present [14]. Active patients refer to those who have not yet recovered and are still COVID-19 positive. This is remarkable as ~ 99.8% cases have recovered and only 0.1% are remaining active. Further, out of the active cases not even a single patient is in critical condition in the hospital currently [14].

We then compared the fatality rates of both SARS-CoV and SARS-CoV-2 to understand how efficiently Singapore managed to learn from the previous outbreaks to strengthen their health care system. The total number of deaths caused by SARS-2003 outbreak in Singapore was 33 out of 238 total cases which makes up to 14% fatality rate (number of deaths/numbers of confirmed cases ×100) in four months (February-May 2003) (**Figure 2****,** Panel B). However, for SARS-CoV-2, from the onset of disease from January to 8 December 2020, there are only 29 documented deaths out of total 58 285 cases which gives fatality rate of only 0.05% in the course of 12 months (January-December 2020). We observed a similar trend for both SARS-CoV and SARS-CoV-2 in which the highest number of deaths occurred in the month of April while no deaths were documented in the initial 2-3 months of infection (**Figure 2****,** Panel B).

Singapore has been able to control the number of deaths caused by COVID-19 and the fatality rate is extremely low as compared to the SARS-2003 outbreak. However, the differences in the infection rate of SARS-CoV and SARS-CoV-2 can be attributed to the fact that in SARS-CoV-2, the number of asymptomatic/pre-symptomatic patients tracing is difficult [15]. It has been noted so far, that these patients affected by SARS-CoV-2 exhibit viral shedding from the upper airway tract in contrast to SARS-CoV, where viral replication predominantly occurred in lower airway tract [16]. In addition, person to person transmission is initiated early during the sub-clinical/ pre-clinical stage in SARS-CoV-2 compared to SARS-CoV [17,18]. These attributes reveal that SARS-CoV-2 has high transmissibility though lower virulence compared to SARS-CoV. These have profound public health implications for testing and containment measures. However, public health systems have to be on alert for the accidental emergence of a new coronavirus that has the worst attributes from both SARS-CoV and SARS-CoV-2: that of high mortality (SARS-CoV) and high transmission (SARS-CoV-2).

A large number of health care workers have been infected in various countries globally but in Singapore only a handful of health care staff have been infected [19]. This was made possible since from the start of COVID-19, health care professionals took all necessary precautions while dealing with infectious patients like wearing N95 masks, hand hygiene, etc.[19]. To effectively trace and slow the spread of SARS-CoV-2, Singapore has initiated handing out Bluetooth-enabled contact tracing devices named as “Trace Together Token” as an alternative to tracing smartphone apps [20]. Moreover, Singapore-developed COVID-19 vaccine called “Lunar-CoV-19” has also been injected into the first batch of volunteers. To enforce social distancing amongst the nationals, Singapore has initiated to deploy pilotless drones for monitoring of distancing norms in crowded areas [21]. Further, Singapore has linked around 99% of the new cases to the known clusters [14]. As of 26 October 2020, Singapore’s testing rate is now among the highest in the world (3 680 000 swabs and 1 078 551 people tested which corresponds to ~ 189 people tested per thousand people) [14]. The government of Singapore also keeps updating the public places visited by COVID-19 cases in the community regularly [14]. This information is regularly updated to inform people to avoid visiting these places and thereby avoid getting infected as well.

## CONCLUSIONS

Singapore’s ability to learn from its past outbreak experiences has given it an edge to build a robust health and surveillance system. Singapore is showing promising results in containing the spread and is an example to the world for their resilience in fighting the current pandemic.
